# Isolated Tuberculosis of the Radius in an Immunocompetent Indian Male Without Pulmonary Involvement: A Report of a Rare Case

**DOI:** 10.7759/cureus.67170

**Published:** 2024-08-19

**Authors:** Sankalp Yadav, Madhan Jeyaraman, Gautam Rawal, Naveen Jeyaraman

**Affiliations:** 1 Medicine, Shri Madan Lal Khurana Chest Clinic, New Delhi, IND; 2 Orthopedics, ACS Medical College and Hospital, Dr MGR Educational and Research Institute, Chennai, IND; 3 Respiratory Medical Critical Care, Max Super Speciality Hospital, New Delhi, IND

**Keywords:** tb, bone tb, radius tb, cartridge based nucleic acid amplification test (cbnaat), mtb (mycobacterium tuberculosis)

## Abstract

Extrapulmonary tuberculosis is less commonly reported, and isolated tuberculous involvement of bones such as the radius, without any pulmonary lesions, is extremely rare. Diagnosing this condition can be challenging due to ambiguous clinical features and non-specific radiological findings in the early stages. The present case describes a rare instance of isolated tuberculosis of the radius in an immunocompetent Indian male with no pulmonary involvement. The diagnosis was achieved through a high index of suspicion in an endemic region, advanced radiometric investigations, and the isolation of *Mycobacterium tuberculosis* using the cartridge-based nucleic acid amplification test. The patient was started on a 12-month course of appropriate chemotherapy.

## Introduction

Tuberculosis is an infectious disease caused by *Mycobacterium tuberculosis*. While the disease predominantly presents with pulmonary symptoms, extrapulmonary tuberculosis is also widely reported in high-burden countries [1]. However, extrapulmonary tuberculosis without pulmonary involvement is exceedingly rare.

Bone tuberculosis occurs in 10-15% of patients with tuberculosis, making it relatively rare [2]. It is more likely to affect bones and joints in older and pediatric patients. While hematogenous dissemination is the primary cause, lymphatic or contiguous spread may also contribute [3,4]. We present a rare case of isolated tuberculosis of the radius, with no pulmonary involvement, in an immunocompetent Indian male.

## Case presentation

A 28-year-old nondiabetic Indian male from a low socioeconomic background presented to the outpatient department with complaints of pain and swelling in his left forearm for one month. The swelling had been insidious in onset and had gradually progressed to its current size over the past 30 days. There was no history of falls, trauma, fever, cough, weight loss, or other signs of tuberculosis.

He worked as a factory worker and had no history of substance abuse or staying in night shelters. Additionally, there was no history of tuberculosis in himself or his contacts, nor had he received any immunosuppressive therapy in the past.

A general examination revealed a hemodynamically stable young male with an ectomorphic build. Local examination showed swelling and tenderness in the middle and distal left forearm, with no discharging sinus or engorged veins. The systemic examination was unremarkable.

**Figure 1:**
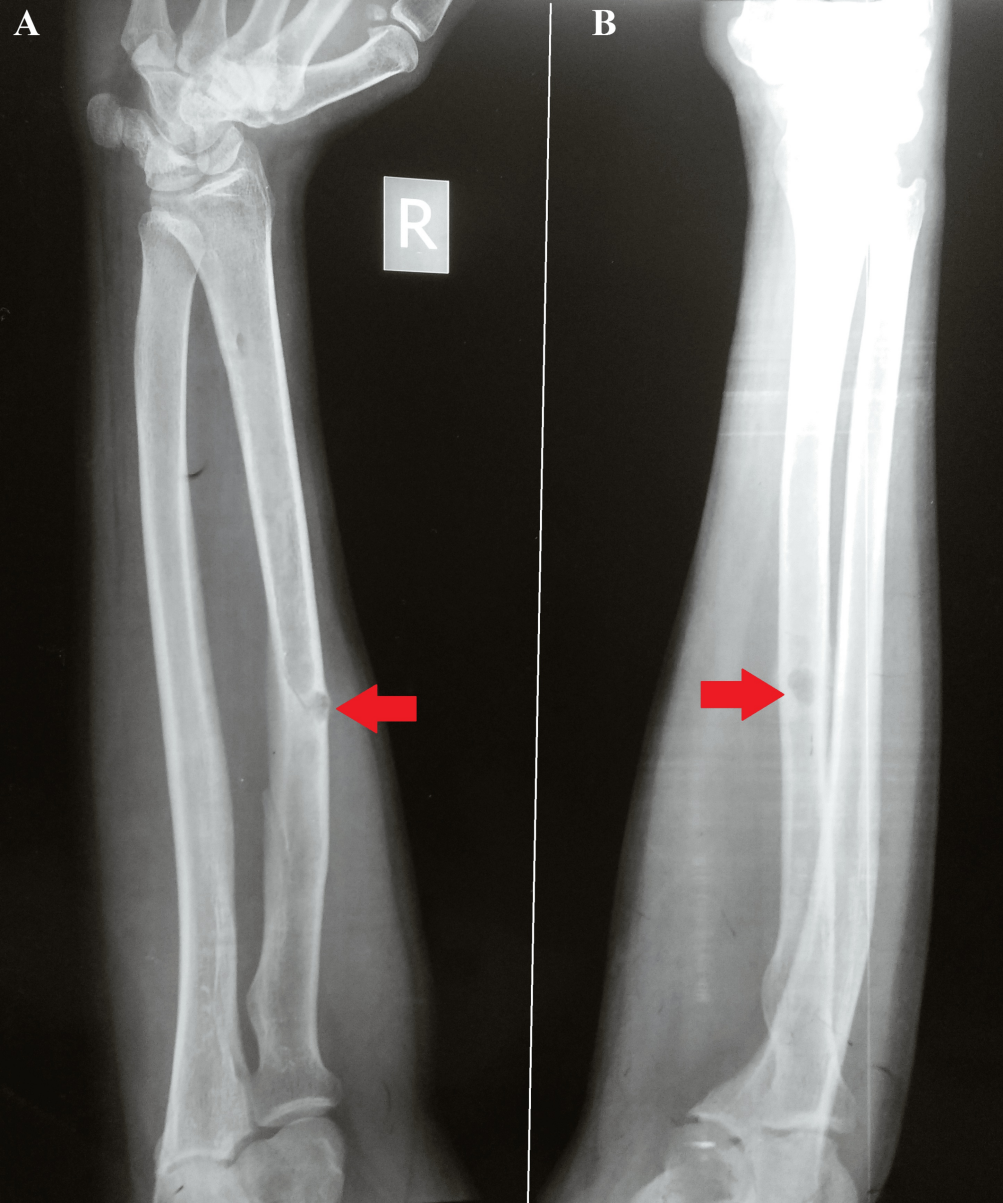
Plain radiograph of the left forearm and the wrist (A) Anteroposterior view. (B) Lateral view. Arrows indicate the lesions in the left radius.

A radiograph of the left forearm and wrist indicated lytic lesions in the left radius (Figure 1).

A chest radiograph was normal. Laboratory tests revealed a marginally elevated erythrocyte sedimentation rate of 25 mm in the first hour. HIV (I and II), rheumatoid factor, induced sputum for acid-fast bacilli, cartridge-based nucleic acid amplification tests, and hepatitis (A, B, and C) were all negative.

**Figure 2:**
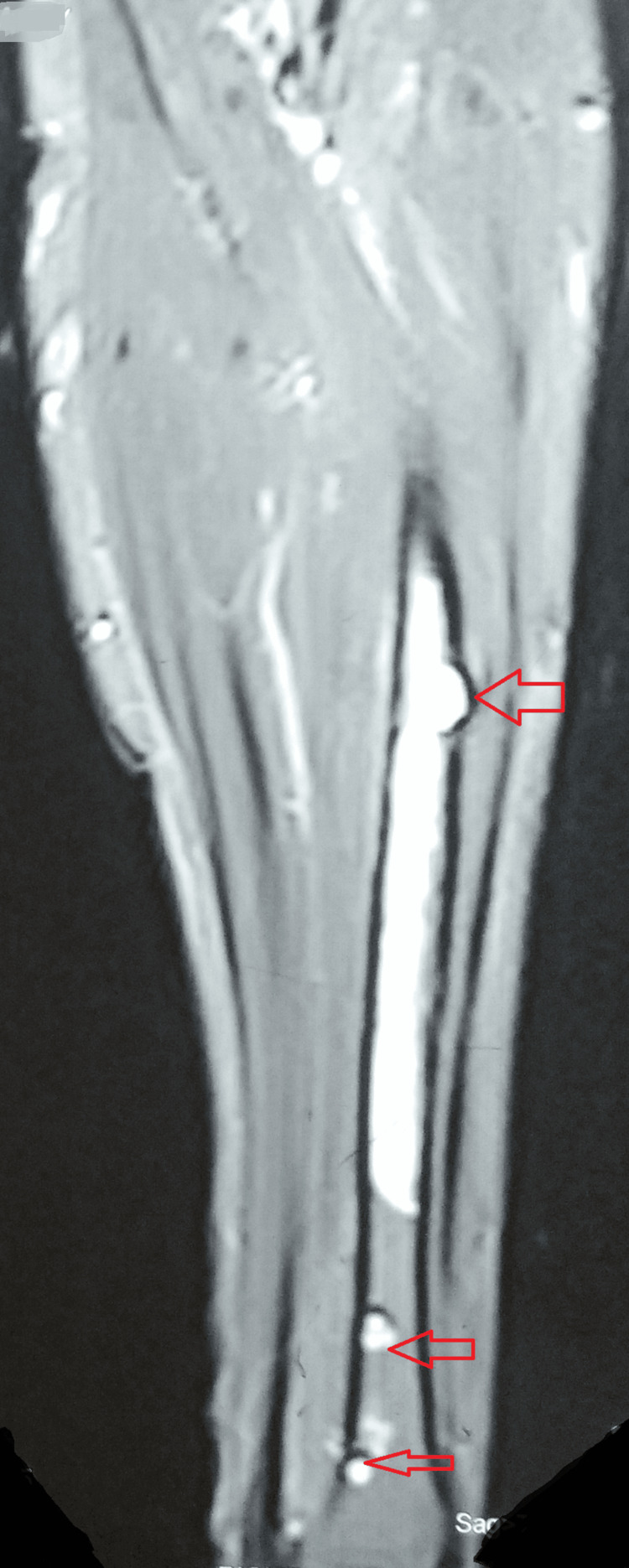
MRI of the left forearm Arrows indicate abnormal hyperintense marrow signals in the mid-shaft of the radius, approximately 11 cm in length. Similar small foci are observed in the distal shaft of the radius.

MRI of the left forearm showed abnormal T2-weighted short-tau inversion recovery hyperintense and T1 hypointense marrow signals in the mid-shaft of the radius, extending approximately 11 cm in length. Similar small foci were observed in the distal shaft of the radius. A focal periosteal reaction was noted in the mid-shaft with minimal overlying edema (Figure 2).

Based on the findings, a biopsy of the lesion in the left radius was performed. Histopathological examination revealed a few Langhans giant cells amid a background of lymphocytes. However, acid-fast bacilli were not detected using the Ziehl-Neelsen stain. Samples were also tested using the cartridge-based nucleic acid amplification test and liquid culture. The nucleic acid amplification test detected very low levels of *Mycobacterium tuberculosis* with no resistance to rifampicin, but the culture was negative by the 48th day.

A diagnosis of tuberculosis of the radius without pulmonary involvement was made. The patient was started on a fixed-dose combination of antituberculous drugs, initially for two months, including isoniazid, pyrazinamide, rifampicin, and ethambutol. Treatment was then continued for an additional 10 months with a fixed-dose combination of isoniazid, rifampicin, and ethambutol. There was significant improvement in his condition, with reduced pain and swelling by the end of the two-month intensive phase. However, at the conclusion of this phase, the patient requested a transfer to a different state for work, which was approved. He was advised on complete treatment adherence and regular follow-ups at the infectious disease and orthopedic clinics. His treatment outcome was recorded as cured in the national data portal Nikshay at the end of 12 months.

## Discussion

Tuberculosis poses a significant public health threat in endemic countries [5]. About 25% of the global population is affected by latent tuberculosis [6]. While tuberculosis is primarily reported as a pulmonary disease, there are documented cases of isolated extrapulmonary involvement, such as in the bones of the upper limb [7]. Osteoarticular involvement occurs in approximately 1-5% of all tuberculosis cases and 10-18% of extrapulmonary cases [8].

Moreover, *Mycobacterium tuberculosis *can spread to any organ in the body after entry through the airways, especially if the immune system is compromised [2]. However, the development of tuberculosis in rare sites, such as the radius, in immunocompetent individuals is seldom reported.

Tuberculosis can affect the entire skeleton, with the spine being the most commonly involved site, while the radius is less frequently affected. Trauma to the affected joint is often noted, and unilateral lesions are a common clinical presentation of bone tuberculosis. Radiographically, the lesions typically show areas of sclerosis surrounding osteolytic lesions with irregular borders. Since these findings can resemble those of pyogenic osteomyelitis, fungal infections, metastasis, telangiectatic osteosarcoma, aneurysmal cysts, sarcoidosis, eosinophilic granuloma, and chordoma, bone lesions with cystic cavities on X-rays are nonspecific [9-11].

The diagnosis is further complicated by the lesions’ location and paucibacillary nature, which provide bacteriological evidence in only about 25% of cases. This makes diagnosing extrapulmonary variants of the disease, such as those affecting the radius, more challenging [2].

Management primarily involves medical treatment with antituberculous drugs according to national guidelines [12]. Surgery is rarely necessary, except in severe cases where bony lesions significantly impact the patient’s quality of life.

A similar case was reported by Lazrek et al. involving a 52-year-old Moroccan woman, with both cases demonstrating involvement of a significant portion of the radius. However, the present case is distinct due to the absence of pulmonary foci, which were present in Lazrek’s case [13]. Additionally, the management of the present case was entirely conservative, in contrast to the approach used in their case.

## Conclusions

Diagnosing tuberculosis in immunocompetent patients without pulmonary involvement is challenging. We present a rare case of tuberculosis of the radius in a 28-year-old nondiabetic Indian male, with no pulmonary involvement. The diagnosis was made through a comprehensive clinical and radiometric workup. Clinicians should consider the possibility of rare presentations of common diseases in endemic areas, as diagnostic difficulties can lead to missed or delayed diagnoses, potentially affecting treatment outcomes. This case underscores the importance of documenting such rare instances of tuberculosis to enhance clinical awareness among primary care providers.

## References

[1] Jilani TN, Avula A, Zafar Gondal A (2024). Active tuberculosis. StatPearls [Internet].

[2] Gomes VM, Dos Santos TC, Cañete LA, Figueira C, Albuquerque R (2019). Tuberculosis of the radius in a child. Radiol Bras.

[3] Gopalaswamy R, Dusthackeer VNA, Kannayan S, Subbian S (2021). Extrapulmonary tuberculosis—an update on the diagnosis, treatment and drug resistance. J Respir.

[4] Santos FC, Nascimento AL, Lira LA, Lima JF, Montenegro Rde A, Montenegro LM, Schindler HC (2013). Bone tuberculosis: a case report on child. Rev Soc Bras Med Trop.

[5] Sulis G, Roggi A, Matteelli A, Raviglione MC (2014). Tuberculosis: epidemiology and control. Mediterr J Hematol Infect Dis.

[6] Haddad MB, Raz KM, Lash TL (2018). Simple estimates for local prevalence of latent tuberculosis infection, United States, 2011-2015. Emerg Infect Dis.

[7] Desdiani D, Rizal H, Basuki A, Fadilah F (2021). Case report: delayed treatment of tuberculosis of the elbow joint. F1000Res.

[8] Yadav S (2024). Spina ventosa of the left second metacarpal in an adult Indian male with no pulmonary involvement: a first-of-its-type case. Cureus.

[9] Sharma R, Gupta P, Mahajan M, Arora M, Gupta A (2017). X-ray and computed tomography findings in macrodystrophia lipomatosa of the foot with secondary osteoarthritic changes diagnosed in an elderly female: a case report. Radiol Bras.

[10] Reis LM, Duarte ML, Alvarenga SB, Prado JL, Scoppetta LC (2018). Sarcoidosis: when the initial manifestations are musculoskeletal symptoms. Radiol Bras.

[11] Costa FM, Canella C, Vieira FG, Vianna EM, Meohas W, Marchiori E (2018). The usefulness of chemical-shift magnetic resonance imaging for the evaluation of osteoid osteoma. Radiol Bras.

[12] (2024). Training module on extrapulmonary tuberculosis. https://tbcindia.mohfw.gov.in/wp-content/uploads/2023/05/7702334778Training_Module_on_Extrapulmonary_TB_-_Book_24032023.pdf.

[13] Lazrek O, Bassir RA, Sabri EM (2018). Tuberculosis of radius diaphysis: case report and review of literature. Int J Mycobacteriol.

